# Vitamin D Deficiency in Children and Adolescents in Saudi Arabia: A Systematic Review

**DOI:** 10.7759/cureus.52040

**Published:** 2024-01-10

**Authors:** Meath S Alhamed, Fahad Alharbi, Abdullatif Al Joher, Sara Dhahry, Albandari A Fallatah, Omar H Alanazi, Jawaher M Almutiri, Saida S Albaradie, Budoor Aziz A Al Enezi, Mashail S Albishi

**Affiliations:** 1 Family Medicine, King Abdulaziz Hospital, Al-Mubarraz, SAU; 2 General Practice, Al Yamamah Hospital, Riyadh, SAU; 3 Pediatrics, Al Yamamah Hospital, Riyadh, SAU; 4 Paediatrics, Al Yamamah Hospital, Riyadh, SAU; 5 Nursing, Al Yamamah Hospital, Riyadh, SAU

**Keywords:** calcium absorption, increased indoor activities, bone health, food fortification, sun exposure, adolesents, vitamin d deficiency

## Abstract

Vitamin D deficiency is a globally recognized health concern, with particular prominence in specific geographies and demographics. Saudi Arabia, with its unique climatic conditions and cultural practices, has been under scrutiny regarding the prevalence of this deficiency, especially among children and adolescents. This systematic review aimed to assess the prevalence of vitamin D deficiency among children and adolescents in Saudi Arabia by compiling and analyzing various studies to offer a comprehensive view of the situation. The comprehensive web search encompassed a range of databases, including Google Scholar and PubMed, to gather studies published between 2012 and 2023. An analysis was conducted on seven studies, totaling 2,429 participants, with each study focusing on various aspects, regions, and cohorts within Saudi Arabia. These studies employed different methodologies, ranging from cross-sectional surveys to randomized clinical trials. The review unveiled an alarming prevalence of vitamin D deficiency in the studied population. On average, around 81.1% of children and adolescents showcased inadequate vitamin D levels. Specific vulnerable groups, such as those with Type 1 diabetes mellitus or asthma, had pronounced deficiencies. Factors influencing these levels ranged from dietary habits, sun exposure, physical activity, and socioeconomic parameters. The compelling evidence from the studies underscores a consistent health issue among the pediatric population in Saudi Arabia that the overwhelming majority of Saudi children and adolescents lack adequate vitamin D. Addressing this widespread deficiency needs a multifaceted approach. Implementing policies that support vitamin D food fortification, encouraging routine screenings, and launching public awareness campaigns about safe sun exposure and diet can play a transformative role in this health crisis.

## Introduction and background

Vitamin D, a fat-soluble prohormone, plays a pivotal role in maintaining skeletal health and calcium homeostasis, and in the modulation of cellular growth, immune function, and inflammation [1]. Historically known for preventing rickets in children and osteoporosis in adults, contemporary studies are now uncovering its significant implications for overall health and chronic disease prevention [2]. Notably, vitamin D deficiency has become a global public health concern affecting both developed and developing countries across all age groups [3]. The significance of this deficiency extends beyond bone health and has potential implications for chronic diseases, including cardiovascular diseases, diabetes, cancers, and autoimmune diseases [4].

Vitamin D is essential for the maintenance of mineral homeostasis and skeletal health [5-8]. The synthesis of vitamin D occurs on the skin. Because most dietary patterns do not include items containing vitamin D on a daily basis, the contribution from food sources is less substantial. Because of this, it is frequently essential to prescribe vitamin D tablets to those who are deficient in the vitamin due to a lack of sun exposure or when there is a decline in the synthesis of vitamin D on the skin (such as in elderly adults) [9,10].

Surprisingly, despite its sunny climate, Saudi Arabia has reported a high prevalence of vitamin D deficiency among its population, encompassing both adults and children [5]. Factors such as increased indoor activities, traditional clothing that limits sun exposure, dietary habits, and possibly genetic factors contribute to this deficiency [6]. In particular, children and adolescents, during their crucial bone-forming years, are at a heightened risk of complications due to insufficient vitamin D levels, which can impact their growth, immune function, and long-term health [7].

The primary role of vitamin D is to facilitate calcium absorption in the gut, ensuring adequate serum calcium and phosphate concentrations to support metabolic functions and bone mineralization, and to prevent hypocalcemic tetany [11]. Vitamin D also impacts bone remodeling by promoting osteoblast and osteoclast activity, ensuring bone health throughout life [12]. Osteoblasts are responsible for building new bone tissue, while osteoclasts are responsible for breaking down and resorbing old bone tissue. Vitamin D helps to stimulate osteoblast activity, leading to the formation of new bone tissue. This is important for maintaining bone density and strength, and for repairing any damage or micro-fractures that may occur in the bones. Additionally, Vitamin D also helps to regulate the activity of osteoclasts, ensuring that they do not become overactive and break down too much bone tissue. This helps maintain the balance between bone formation and resorption, which is essential for healthy bone remodeling [12].

Vitamin D has been associated with a range of health benefits beyond its traditional role in bone health [13]. Research has indicated that vitamin D may have a role in reducing the risk of certain chronic diseases such as multiple sclerosis, rheumatoid arthritis, and certain types of cancer [14]. Additionally, the presence of vitamin D receptors in immune cells suggests that it may play a role in modulating immune responses, which has led to investigations into its potential impact on infectious diseases, autoimmune conditions, and inflammatory processes. This broader potential role of vitamin D in health and disease prevention is an active area of research and continues to be studied [15].

Observational studies have indicated potential associations between vitamin D deficiency and an increased susceptibility to acute respiratory tract infections, cardiovascular diseases, and neuropsychiatric disorders. However, it's important to note that these associations do not necessarily imply causation and further research is needed to establish the causal nature of these relationships. The potential impact of vitamin D on these health conditions is an area of ongoing investigation, and more research is needed to fully understand the role of vitamin D in these contexts [16].

The purpose of conducting this review is to provide a comprehensive and current analysis of the prevalence, treatment strategies, and implications of vitamin D deficiency, particularly in the pediatric and adolescent population of Saudi Arabia. By synthesizing the latest research and evidence, we aim to offer valuable insights into the extent of this health concern, the effectiveness of various treatment methods, and the potential impact on the overall well-being of young individuals. This review seeks to contribute to the existing body of knowledge, inform healthcare professionals, policymakers, and stakeholders, and ultimately guide the development of targeted interventions and public health initiatives to address and mitigate the effects of vitamin D deficiency in this specific demographic.

## Review

Method

This was a systematic review conducted in September 2023 following the Preferred Reporting Items for Systematic Reviews and Meta-Analyses (PRISMA) guidelines. To retrieve relevant research, a thorough search was conducted across major databases, using PubMed mainly as a search engine for studies. Only studies published in the English language were included. The following keywords were converted into PubMed Medical Subject Headings (MeSH) terms and used to find studies that were related: vitamin D, vit D deficiency, children, adolescents, and Saudi Arabia. The Boolean operators "OR" and "AND" matched the required keywords. 

We considered the following criteria for inclusion in this review: studies that investigated the prevalence of Vitamin D deficiency in Saudi Arabia, clinical trials that investigated Vitamin D deficiency management in Saudi Arabia, and studies that included patients under 18 years old. We excluded studies published before 2012, case reports, and letters to the editors.

Data Extraction and Synthesis

Duplicates in the search strategy output were found using Rayyan software [17]. To determine the relevance of the titles and abstracts, the researchers used a set of inclusion/exclusion criteria to filter the combined search results. The reviewers then analyzed the data and synthesized the findings from the included papers. They also assessed the quality of the studies and considered any potential biases. The authors' thorough approach to data extraction and analysis ensures that the review is based on a comprehensive and rigorous assessment of the available evidence. This approach also helps to ensure that the conclusions drawn from the review are well-supported and reliable.Summary tables were created using information from pertinent research to give a qualitative overview of the results and study components.

Risk-of-Bias Assessment

Using the ROBINS-I (Risk Of Bias In Non-randomised Studies - of Interventions) risk-of-bias assessment approach for non-randomized trials of therapies, the quality of the included studies was assessed [18]. The seven themes that were assessed were confounding, participant selection for the study, classification of interventions, deviations from intended interventions, missing data, assessment of outcomes, and choosing of the reported result.

Results

**Figure 1:**
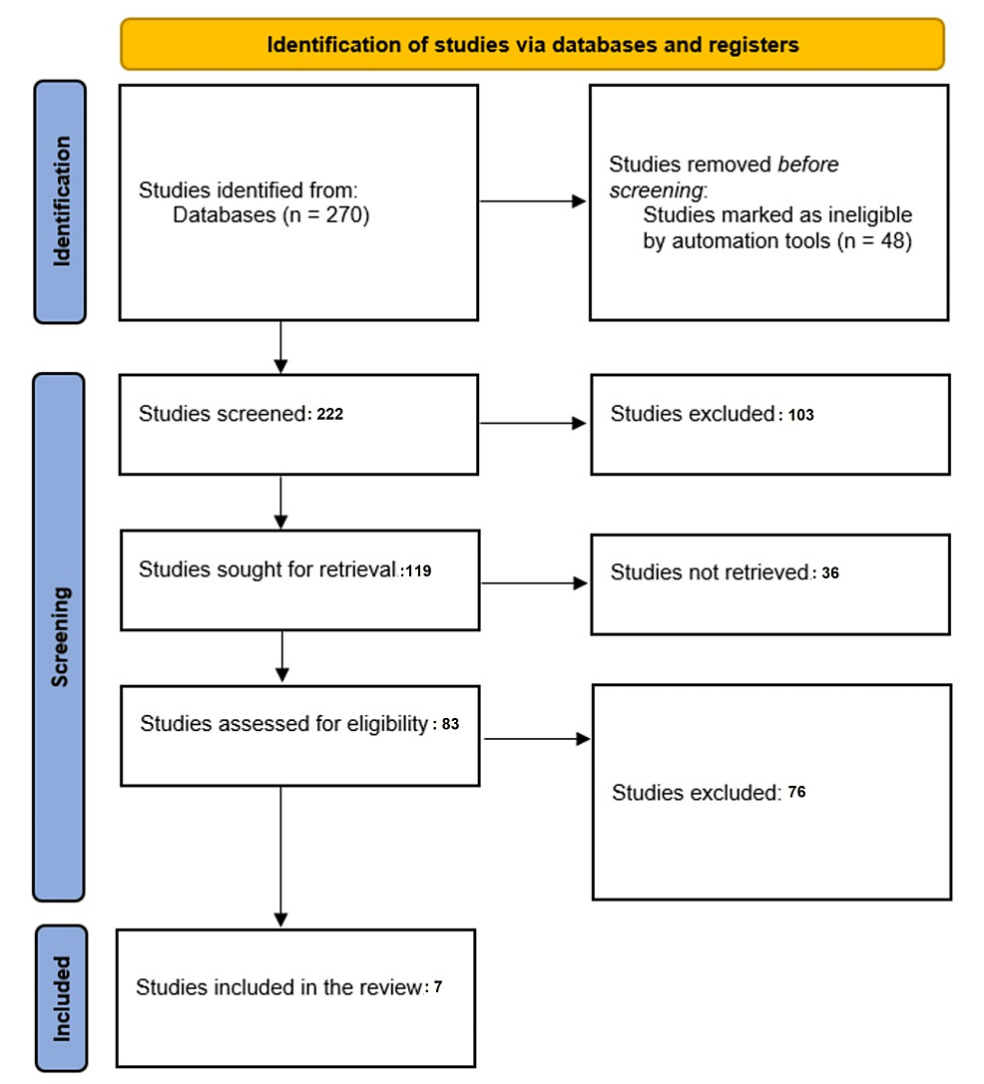
PRISMA flowchart summarizes the study selection process. PRISMA: Preferred Reporting Items for Systematic Reviews and Meta-Analyses

A total of 270 study articles resulted from the systematic search, and 48 were removed because they didn't fit the criteria. Title and abstract screening were conducted on 222 studies, and 103 studies were excluded. A total of 119 studies were sought for retrieval, and only 83 articles were retrieved. Finally, 83 studies were screened for full-text assessment, of which 76 were excluded for either having inappropriate study methodology or results. Seven eligible articles were included in this systematic review. A summary of the study selection process is presented in Figure 1. 

Characteristics of the Included Studies

Table 1 showcases the demographics of studies that focused on vitamin D, detailing study design, location, participant count, age distribution, and gender ratios. The summarized data encompasses seven distinct studies, yielding a total participant count of 2,429 [19-25]. When exploring gender distribution, it's notable that Al-Musharaf et al. [19] reported a 46.2% male participation rate, while Al-Agha et al. [20] presented a slightly higher male population at 50.5%. Al-Hussaini et al. [21] did not specify the male percentage, but Al-Ghamdi et al. [22] recorded a 44.4% male ratio. Bindayel [23] exhibited a 55% male representation and Al-Othman et al. [24] matched Al-Musharaf's findings with a 46.2% male distribution. Lastly, Talaat et al. [25] indicated a slight majority in male composition at 49.3%.

**Table 1:** Sociodemographic characteristics of the included participants.

Study	Study design	Location	Participants	Age range (mean) in years	Males (%)
Al-Musharaf et al., 2012 [19]	Randomized cross-sectional study.	Saudi Arabia (Urban areas)	331	6-17	46.2%
Al-Agha et al., 2016. [20]	Cross-sectional study.	Jeddah	378	9.5 ± 3.9	50.5%
Al-Hussaini et al., 2022. [21]	Cross-sectional mass screening study.	Saudi Arabia (National)	575	6-16	-
Al-Ghamdi et al., 2017 [22]	Cross-sectional study	Albaha	117	1-18	44.4%
Bindayel, 2021 [23]	Cross-sectional study	Riyadh	60	8.1 ± 3.4	55%
Al-Othman et al., 2012 [24]	Cross-sectional study.	Saudi Arabia	331	6-17	46.2%
Talaat et al., 2016 [25]	Randomized, controlled clinical trial.	Taif	637	8.51 ± 3.527	49.3%

These studies were located primarily within Saudi Arabia. Specific cities or regions of focus include urban areas [19], Jeddah [20], and a national scope [21]. Other regions or cities of study encompass Albaha [22], Riyadh [23], and Taif [25]. In terms of the study design, the majority adopted a cross-sectional approach [19-24], while one study [25] utilized a randomized, controlled clinical trial.

Finally, diving into age specifications, the main age group that was targeted by these studies are children and adolescents (aged 2-19 years). Al-Musharaf et al. [19] and Al-Othman et al. [24] focused on participants aged between 6-17 years. Al-Agha et al. [20] reported an average participant age of 9.5±3.9 years, whereas Al-Hussaini et al. [21] concentrated on the age group of 6-16 years. Al-Ghamdi et al. [22] covered a broader age bracket from one to 18 years. Bindayel [23] documented participants with an average age of 8.1±3.4 years, and Talaat et al. [25] reported an average age of 8.51±3.527 years.

Table 2 presents the clinical characteristics summary of the included studies. The studies conducted by Al-Musharaf et al. [19] and Al-Othman et al. [24] shed light on the prevalence of vitamin D deficiency among Saudi children and adolescents. While Al-Musharaf's focus was on urban areas and the impact of dietary calcium intake, Al-Othman examined the relationship between deficiency and physical activity as well as sun exposure. Both studies revealed a widespread prevalence of vitamin D deficiency among the subjects. Al-Musharaf emphasized the significance of fortifying the Saudi diet, while Al-Othman identified the influence of sun exposure and physical activity, ultimately concluding that even individuals with maximum sun exposure were still deficient. These findings underscore the pressing need for comprehensive strategies to address and mitigate the prevalence of vitamin D deficiency in this demographic.

**Table 2:** Clinical characteristics and outcomes of the included studies.

Study	Methodology & Objective	Results	Outcomes/Conclusion
Al-Musharaf et al., 2012 [19]	This study aimed to determine the prevalence of vitamin D deficiency among urban Saudi children and adolescents and its relationship with childhood obesity and dietary calcium intake.	Vitamin D deficiency was observed in all subjects, with females having significantly lower vitamin D levels than males. Mean calcium intake was 60% of RDA and mean vitamin D intake was 23% of RDA. There was no significant difference in vitamin D status and calcium intake between normal and overweight/obese participants.	The findings highlight the importance of vitamin D fortification and increased dietary calcium in the Saudi diet to meet RDA requirements and prevent vitamin D deficiency-related diseases
Al-Agha et al., 2016 [20]	The study aimed to explore the relationship between Vitamin D deficiency and socioeconomic factors in Jeddah, Saudi Arabia	Only 1.9% of the children had normal Vitamin D levels. Lower-income families displayed higher mean Vitamin D levels compared to families with medium and high incomes. A family's size and the father's education level were also associated with differing Vitamin D levels, but the mother's education level was not a determinant.	Lower-income families had the highest mean Vitamin D levels. Vitamin D levels did not show significant correlation with family size or parents’ educational levels.
Al-Hussaini et al., 2022 [21]	The study sought to update the prevalence of micronutrient deficiencies in the Saudi pediatric population and to investigate any association with undernutrition.	Most participants displayed deficiencies in vitamin D (78%) and iron (20%). There were no significant differences in micronutrient levels when comparing thin children to their normal BMI counterparts.	No association was found between undernutrition and micronutrient deficiencies.
Al-Ghamdi et al., 2017 [22]	The study aimed to determine the prevalence of vitamin D deficiency among Saudi children and adolescents with T1DM	Most of the participants (88.9%) displayed vitamin D inadequacy. Girls and younger age groups exhibited a higher prevalence of vitamin D deficiency.	Vitamin D deficiency is prevalent among Saudi children and adolescents with T1DM
Bindayel, 2021 [23]	The study aimed to evaluate the levels of 25-hydroxyvitamin D3 in children with asthma in Riyadh, assessing it in relation to anthropometrics, dietary habits, and sun exposure.	Over 50% of the children displayed insufficient vitamin D levels. Levels were negatively correlated with age, weight, and height. Sun exposure, especially involving multiple body parts, was associated with higher vitamin D levels.	Recommendations include considering vitamin D screening, especially in older and overweight children with asthma.
Al-Othman et al., 2012 [24]	The study aimed to understand the prevalence of vitamin D deficiency in relation to physical activity and sun exposure among Saudi children and adolescents	All subjects had a vitamin D deficiency with 71.6% being moderately deficient. Age was the most significant predictor of vitamin D levels. Physical activity and sun exposure both influenced vitamin D levels, but the highest levels were still deficient.	Vitamin D deficiency is influenced by sun exposure and physical activity. Even those with the most sun exposure and activity are deficient, indicating a need for vitamin D supplementation.
Talaat et al., 2016 [25]	The study assessed the effects of three different vitamin D replacement regimens on levels of 25(OH)D, calcium, and PTH over a year in a pediatric and adolescent population.	There was a significant difference in 25(OH)D levels between the three groups after 4 and 12 months. Groups 2 and 3 saw substantial increases in 25(OH)D after 4 months, but levels dropped in groups 1 and 2 after 12 months. Group 3 continued to show an increase in 25(OH)D levels over the year.	For addressing vitamin D insufficiency, it's advisable to use a low-loading dose followed by a higher maintenance dose. This strategy yields a steady rise in serum 25(OH)D without hypercalcemic side effects.

Al-Agha et al. [20] and Al-Hussaini et al. [21] explored the socioeconomic and nutritional aspects, respectively. Al-Agha's research from Jeddah showed that most children had vitamin D deficiency, interestingly highlighting that lower-income families had higher mean Vitamin D levels. Al-Hussaini, on the other hand, found no association between undernutrition and micronutrient deficiencies.

Al-Ghamdi et al. [22] and Bindayel [23] evaluated vitamin D levels among children with specific health conditions. Al-Ghamdi's research indicated that vitamin D deficiency is prevalent among Saudi children with Type 1 diabetes mellitus (T1DM). Bindayel focused on children with asthma in Riyadh, finding that over half had insufficient vitamin D levels, which correlated negatively with age, weight, and height. Sun exposure, in this case, did seem beneficial, emphasizing the need for vitamin D screening for older, overweight asthmatic children.

The study by Talaat et al. provides valuable insights into the effects of different vitamin D replacement regimens on children in Saudi Arabia [25]. The findings indicating varying results based on the regimen, with a notable increase in vitamin D levels in certain groups, suggest the importance of tailoring replacement regimens to individual needs. The recommendation to start with low-loading doses and then transition to higher maintenance doses aligns with the idea of personalized treatment and could have significant implications for clinical practice. This study's results may inform the development of more effective strategies for managing and preventing vitamin D deficiency in children, ultimately contributing to improved health outcomes in this population [25].

Prevalence of Vitamin D Deficiency in the Studied Population

Studies from various locations across Saudi Arabia have consistently demonstrated a significant prevalence of vitamin D deficiency among children and adolescents. The research by Al-Musharaf et al. in urban areas of Saudi Arabia indicated that all the subjects (100%) were vitamin D deficient [19]. In a related vein, a study from Jeddah by Al-Agha et al. found that a staggering 98.1% of children had inadequate Vitamin D levels [20]. Al-Hussaini et al. updated these findings, revealing that 78% of participants across the nation were deficient in vitamin D [21].

Diving deeper into specific populations, Al-Ghamdi et al. found that 88.9% of Saudi children and adolescents with T1DM showed a lack of adequate vitamin D [22]. Bindayel's research among asthmatic children in Riyadh showed that over 50% had insufficient vitamin D levels [23]. Al-Othman et al. added more granularity to these findings by showing that while all their subjects had a vitamin D deficiency, 71.6% were moderately deficient [24].

On averaging the deficiency percentages from the mentioned studies: 100% [19], 98.1% [20], 78% [21], 88.9% [22], 50% [23], and 71.6% [24], we get an overall average prevalence of about 81.1% for Vitamin D deficiency across these studies in Saudi Arabia. This figure underscores the urgency of addressing this health issue among children and adolescents in the region.

Discussion

This systematic review included various studies centered on vitamin D deficiency in Saudi Arabian children and adolescents, with a combined participant count of 2,429 [19-25]. The gender distribution varied slightly across the studies, but overall, the male representation hovered around the 50% mark. Geographically, these studies spanned different regions within Saudi Arabia, from urban areas to specific cities, emphasizing the widespread nature of this health concern. Most of the studies employed a cross-sectional approach, aiming to capture a snapshot of vitamin D levels in their chosen demographics. The age demographics centered around children and adolescents, emphasizing the criticality of addressing this health issue in the formative years. The implications of the results are multifaceted. Firstly, the combined participant count of 2,429 from the various studies provides a substantial sample size, enhancing the robustness and generalizability of the findings. The gender distribution hovering around the 50% mark for males indicates that both male and female representation in the studies was fairly balanced, allowing for a comprehensive understanding of the prevalence of vitamin D deficiency across genders. The geographic diversity of the studies, spanning different regions within Saudi Arabia, underscores the widespread nature of the health concern, emphasizing the need for a nationwide approach to address this issue. The predominance of cross-sectional approaches in the studies highlights the need for longitudinal research to better understand the trends and long-term implications of vitamin D deficiency in this population. Furthermore, the focus on children and adolescents in the age demographics underscores the criticality of addressing vitamin D deficiency during the formative years. Early intervention and management of vitamin D deficiency in this age group can have long-term implications for their overall health and well-being as they transition into adulthood.

Studies across Saudi Arabia have persistently highlighted a profound prevalence of vitamin D deficiency in children and adolescents, with research from urban areas to specific cities reflecting alarmingly high deficiency rates. For instance, Al-Musharaf et al. [19] reported a complete deficiency in their urban study population, while Al-Agha et al. [20] from Jeddah and Al-Hussaini et al. [21] found 98.1% and 78% deficiency rates, respectively. When considering specific health conditions, Al-Ghamdi et al. [22] observed that 88.9% of children with T1DM were vitamin D deficient, while over half of the asthmatic children in Bindayel's study [23] faced a similar challenge. Collectively, these findings average out to an 81.1% deficiency rate, emphasizing the critical need for interventions in the pediatric and adolescent population of Saudi Arabia. The 81.1% deficiency rate in the pediatric and adolescent population of Saudi Arabia is notably high compared to global averages. According to the World Health Organization, this rate surpasses the global average of vitamin D deficiency, which is approximately 30-50% in children and adolescents worldwide. This emphasizes the urgent need for interventions and public health initiatives to address this significant health concern in Saudi Arabia.

The study by Al-Musharaf et al. puts the prevalence of this deficiency into sharp relief, with every participant displaying signs of inadequate vitamin D levels [19]. This deficiency persisted across both genders, although girls were at a higher disadvantage. Remarkably, factors such as obesity or the dietary intake of calcium did not appear to influence this deficiency significantly, though there was a notable link between higher calcium intake and improved vitamin D status. This underscores the need for a comprehensive approach to nutritional education and supplementation in the country.

Looking at global research on both adults and children indicates that vitamin D insufficiency is commonly seen in those who are overweight or obese. The exact reason for this deficiency in obese individuals remains uncertain. One theory suggests that vitamin D might get trapped in subcutaneous fat, reducing its bioavailability in the body. Another perspective points to the possibility that obese individuals might spend less time outdoors, leading to decreased sunlight exposure and, consequently, reduced natural vitamin D production. In adults, low levels of vitamin D have been linked to the onset of cardiovascular diseases. A recent extensive study revealed that being overweight during the teenage years could elevate the risk of specific diseases in later life. The observed vitamin D deficiency in overweight adolescents might be a factor influencing this increased risk [26-30].

The socioeconomic analysis presented by Al-Agha et al. offers an interesting perspective [20]. While vitamin D deficiency remained prevalent, it was paradoxically more common among medium to high-income families. This could indicate that certain lifestyle factors associated with higher incomes, such as spending more time indoors or using sunscreen regularly, might contribute to these deficiencies. However, a lack of correlation between vitamin D levels and factors like family size or parental education suggests a more nuanced interplay of socioeconomic factors.

Al-Hussaini et al.'s study in 2022 provided a comprehensive micronutrient assessment, reaffirming the significant rates of vitamin D and iron deficiencies [21]. However, it's encouraging to note that deficiencies in other essential trace elements, such as zinc, copper, and selenium, are not as pronounced. Still, the absence of a connection between undernutrition and these micronutrient deficiencies suggests that vitamin D deficiency is a distinct issue that requires targeted interventions.

The study conducted by Al-Ghamdi et al. is particularly concerning given the already vulnerable health status of the cohort studied [22]. Children and adolescents with T1DM already grapple with the challenges of their condition, and the compounded effect of a significant vitamin D deficiency adds another layer of health risk. The heightened deficiency in girls and younger children further underlines the need for targeted interventions [26].

Vitamin D intake during pregnancy and early childhood might lower the risk of T1DM. In a study of children with a higher genetic risk for this condition, those whose mothers consumed more vitamin D from food had a reduced likelihood of developing early signs of diabetes, although prenatal vitamin D supplements didn't show this protective effect [27]. Another long-term study on Finnish children revealed that consistent intake of a specific dose of vitamin D during infancy greatly reduced their chances of developing T1DM [31]. However, a Swedish study provided contrasting results and showed that while vitamin D supplementation in pregnant mothers might offer some protection for their infants at a year old, this effect wasn't evident by the age of 2.5 years, and daily vitamin D supplements during infancy didn't have a significant impact [32]. The inconsistent results across these studies might arise from varying vitamin D dosages and the absence of comparative data on maternal vitamin D levels. Additionally, the use of different testing methods for vitamin D across these studies complicates direct comparisons [31-34].

Bindayel's 2021 study delves deep into the realm of asthmatic children in Riyadh and paints a vivid picture of vitamin D deficiency in this group [23]. While it's intriguing that younger participants showed a higher level of vitamin D sufficiency, it remains alarming that children with higher BMI, often linked with lesser physical activity and poor dietary habits, exhibited more pronounced deficiencies. The dietary findings from the study hint at a possible pathway for intervention; by promoting regular consumption of vitamin D-rich foods such as fish, eggs, and liver, it might be possible to curtail the deficiency rates. Furthermore, the association between sun exposure to multiple body parts and higher vitamin D levels is a testament to the age-old understanding of sunlight being a primary source of vitamin D. As such, advocating for safe sun exposure practices, especially in a region like Riyadh, which is endowed with abundant sunlight, could be pivotal.

In the study by Al-Othman et al., it's disconcerting to see that all participants, irrespective of their levels of physical activity or sun exposure, had some degree of vitamin D deficiency [24]. This ubiquity of deficiency suggests that there might be other systemic or external factors at play beyond the traditional culprits of inadequate sun exposure and physical inactivity. While these elements do influence the vitamin D status, as evidenced by physically active subjects and those with regular sun exposure having relatively better levels, the fact remains that even these groups were deficient. This observation nudges toward the direction that mere lifestyle alterations might not be enough, but more direct interventions might be imperative.

Vitamin D insufficiency is often more common among girls. In a study of Turkish adolescent girls, the rate of vitamin D insufficiency varied significantly based on socio-economic status and season [26]. In another study, girls displayed a higher prevalence of vitamin D deficiency [35]. This trend was observed even in older age brackets. Notably, nearly two-thirds of the girls aged between 10 and 16 years had low vitamin D levels. Potential reasons for this include traditional clothing habits among Turkish girls and limited outdoor activities. Recent research indicates that young women who wear more covering attire often have vitamin D deficiency and might be at a heightened risk for conditions like osteoporosis [26,36-40].

The study by Talaat et al. is an excellent illustration of a targeted intervention to combat vitamin D insufficiency [25]. By exploring three distinct vitamin D replacement regimens, the study provides practical insights that can be applied in clinical settings. The consistent increase in 25(OH)D levels in group 3 across the year signifies the efficacy of a regimen that combines a low-loading dose with a higher maintenance dose. This nuanced understanding can guide healthcare professionals in tailoring vitamin D supplementation strategies for children and adolescents, ensuring not just an initial spike but a sustained improvement in vitamin D levels.

The best method and duration of vitamin D treatment for deficiencies in infants and children is still a matter of debate, with guidance largely based on findings from limited studies. A prevalent recommendation is the use of vitamin D2 or D3 for a span of 12 weeks at dosages ranging from 1,000-2,000 IU daily for infants, and as much as 4,000 IU daily for children over one year [33]. This approach aims for a total cumulative dose between 200,000 and 600,000 IU. There are alternative treatment methods, such as monthly intramuscular injections ranging from 10,000 to 50,000 IU spanning three to six months [41], or a single oral dose between 300,000-600,000 IU [42]. For older children and adults, a suggested cost-effective treatment for vitamin D deficiency is an oral dose of 50,000 IU of vitamin D2 once a week for two months [43], which then transitions to a maintenance dose taken every two to four weeks. Some experts recommend a consistent dosage of 400 IU of vitamin D over six months to a year to sustain vitamin D levels post treatment [44].

Together, these studies are a testament to the multifaceted nature of the vitamin D deficiency conundrum. Addressing this requires a holistic approach that considers not just dietary and lifestyle habits but also targeted clinical interventions, especially in populations or cohorts more vulnerable to deficiency, such as children with specific health conditions like asthma or T1DM.

This review offers valuable insights into the prevalence and implications of vitamin D deficiency in this specific population. However, it is important to acknowledge its limitations in order to provide a comprehensive understanding of its findings. One limitation is the potential for bias in the selection of included research articles. The systematic review process relies on the availability and quality of existing literature, which may introduce a certain degree of bias in the findings. Additionally, the study may be limited by the quality and consistency of the data available in the selected articles, which could impact the overall reliability of the conclusions. Furthermore, the study may be constrained by the scope of the research and the specific demographic focus on children and adolescents in Saudi Arabia. This narrow focus may limit the generalizability of the findings to other populations or age groups. Additionally, the study may be limited by the lack of long-term follow-up data, which could provide further insights into the implications of vitamin D deficiency over time.

## Conclusions

The body of research clearly establishes the urgency to address vitamin D deficiency in Saudi Arabia's pediatric and adolescent population. While different studies focus on various cohorts and perspectives, the overarching narrative remains consistent: a substantial portion of Saudi children and adolescents are not receiving adequate vitamin D. Addressing this deficiency is not just about immediate health outcomes; it's about ensuring a healthier future for the country's next generation. The introduction of policies promoting vitamin D fortification in foods, widespread screenings, and public awareness campaigns about the importance of sun exposure and dietary choices could be pivotal in reversing this trend.
